# Inferring circRNA-drug sensitivity associations via dual hierarchical attention networks and multiple kernel fusion

**DOI:** 10.1186/s12864-023-09899-w

**Published:** 2023-12-21

**Authors:** Shanghui Lu, Yong Liang, Le Li, Shuilin Liao, Yongfu Zou, Chengjun Yang, Dong Ouyang

**Affiliations:** 1 Faculty of Innovation Enginee, Macau University of Science and Technology, Avenida Wai Long, Taipa, 999078 Macao, Macao Special Administrative Region of China China; 2 School of Mathematics and Physics, Hechi University, No.42, Longjiang, 546300 Guangxi, China; 3 Peng Cheng Laboratory, Shenzhen, 518055 Guangdong, China; 4 School of Artificial Intelligence and Manufacturing, Hechi University, No.42, Longjiang, 546300 Guangxi, China

**Keywords:** circRNA-drug sensitivity associations, Multimodal networks, Bi-typed multi-relational heterogeneous graphs, Attention mechanism, Multi-kernel fusion

## Abstract

Increasing evidence has shown that the expression of circular RNAs (circRNAs) can affect the drug sensitivity of cells and significantly influence drug efficacy. Therefore, research into the relationships between circRNAs and drugs can be of great significance in increasing the comprehension of circRNAs function, as well as contributing to the discovery of new drugs and the repurposing of existing drugs. However, it is time-consuming and costly to validate the function of circRNA with traditional medical research methods. Therefore, the development of efficient and accurate computational models that can assist in discovering the potential interactions between circRNAs and drugs is urgently needed. In this study, a novel method is proposed, called DHANMKF , that aims to predict potential circRNA-drug sensitivity interactions for further biomedical screening and validation. Firstly, multimodal networks were constructed by DHANMKF using multiple sources of information on circRNAs and drugs. Secondly, comprehensive intra-type and inter-type node representations were learned using bi-typed multi-relational heterogeneous graphs, which are attention-based encoders utilizing a hierarchical process. Thirdly, the multi-kernel fusion method was used to fuse intra-type embedding and inter-type embedding. Finally, the Dual Laplacian Regularized Least Squares method (DLapRLS) was used to predict the potential circRNA-drug sensitivity associations using the combined kernel in circRNA and drug spaces. Compared with the other methods, DHANMKF obtained the highest AUC value on two datasets. Code is available at https://github.com/cuntjx/DHANMKF.

**Supplementary Information:**

The online version contains supplementary material available at 10.1186/s12864-023-09899-w.

## Introduction

Circular RNA (circRNA) is a unique type of RNA that differs from other RNAs in that it forms a covalently closed loop and is typically considered non-coding. With the advancement of high-throughput genomics technology, circRNA has become a hot topic in RNA biology research [1]. Since the discovery of the first circRNA in RNA viruses in the 1970s [2], the advancement of biomedical technology has resulted in the discovery of an increasing amount of circRNAs. However, research into circRNA function has progressed very slowly over several decades, until 2013, when Memczak et al. and Hansen et al. proved that the circular RNA of human cerebellar degeneration-related protein has an important function in neural development [3, 4]. This discovery led to a great increase in the study of circRNA function. The most notable function of circRNAs is that they act as miRNA sponges, which regulates target gene expression by inhibiting miRNA activity. One circRNA can regulate one or multiple miRNAs through multiple miRNA binding sites in a circular sequence [5]. Previous studies have found that circRNA can regulate alternative splicing or transcription [6, 7], as well as parental gene expression [8, 9]. The results of these studies have also indicated that circRNA plays an important role in physiological and pathological processes, and that the dysregulation of circRNA is closely related to many human diseases [10]. over the past two decades, several verified biological function experiments have shown that circRNA has potential as a new clinical diagnostic marker.

Over the years, an increasing number of studies have demonstrated that circRNA can significantly affect the drug sensitivity of cells. For example, Gao et al. [11] screened 18 circRNAs from 3093 circRNAs and then verified them in real-time by quantitative reverse transcription PCR. Finally, hsa_circ_0006528 was found to play an important role in chemotherapy resistance in breast cancer patients. Peng et al. [12] first used next-generation sequencing (NGS) technology to identify the comprehensive circRNA expression profile of multi-drug-resistant osteosarcoma(OS) cell lines and found that hsa_circ_ 0004674 was significantly elevated in OS-resistant cells and tissues, and was associated with poor prognosis. This was then verified by quantitative real-time PCR (qRT-PCR). A study by Wu et al. [13] found that hsa_circ_0001546 is decreased in gastric cancer, which is associated with poor prognosis and also inhibits drug resistance via the ATM/Chk2/p53-dependent pathway. Ruan et al. [14] used four identification algorithms to describe the expression profile of circRNA in approximately 1000 human cancer cell lines and observed a strong correlation between circRNA expression and drug response. That study systematically demonstrated the effect of circRNAs on drug sensitivity. However, research into the relationship between circRNA and drug sensitivity is a newly emerging field that has developed rapidly over the past decade, so our understanding of this relationship is still in its early stages.

The process of validating the relationships between circRNA and drug sensitivity using traditional biomedical methods is time-consuming and costly. Therefore, some researchers have developed computational models that can help to reveal the potential relationships between circRNA and drugs. For example, Deng et al.[15] proposed a computational model called GATECDA for predicting the association between circRNA and drug sensitivity. GATECDA is based on the Graph Attention Auto-encoder(GATE) [16]. First, sequence information data for circRNAs, structural data for drugs, and circRNA-drug sensitivity association data were collected. Then the similarity between circRNAs and drugs were each calculated and these data as well as circRNA-drug sensitivity association data were input into the GATE, in order to generate low-dimensional vector representations of circRNA and drug nodes. Finally, the low-dimensional vector representations generated by the GATE were input into a fully connected neural network for circRNA-drug sensitivity association prediction. Later, Yang et al. [17] proposed a model called MNGACDA. The model constructs a multimodal network based on multiple information sources on circRNAs and drugs. Then, a node-level attention Graph Auto-Encoder was used to obtain low-dimensional embeddings of circRNAs and drugs from the multimodal network. Finally, the low-dimensional embeddings of circRNAs and drugs were input into an inner product decoder to score the association between circRNAs and drug sensitivity. To our knowledge, these were the first models to apply computational methods to predict the potential association between circRNAs and diseases. Thus far, no other new models have been applied in this field, and considerable advancement in the creation of new and improved models for this field of research is much needed.

Since Multiple Kernel Learning (MKL) [18] was proposed, it has been widely applied to bipartite biological networks for the improvement of model performance. Specifically, the information contained in the samples were used by MKL to compute the multiple kernel matrix, and then the optimal kernel matrix was obtained by fusing multiple kernel matrices. For example, MKGCN, which is based on MKL and GCN [19], was proposed by Yang et al. to infer novel microbe-drug associations. Yan et al. [20] proposed a computational methods called MKLC-BiRW, based on MKL and Bi-random walk algorithm, to predict potential drug-target interactions by integrating diverse drug-related and target-related heterogeneous information.

In this study, we propose a novel method, called DHANMKF, that aims to predict potential circRNA-drug sensitivity associations for further biomedical screening and validation. Firstly, multimodal networks were constructed by DHANMKF using multiple sources of information on circRNAs and drugs. Secondly, comprehensive intra-type and inter-type node representations were learned using multi-relational heterogeneous graphs, which are attention-based encoders under a hierarchical process. Thirdly, a multi-kernel fusion method was used to fuse intra-type embedding and inter-type embedding. Fourthly, the Dual Laplacian Regularized Least Squares (DLapRLS) method was used to predict the potential circRNA-drug sensitivity associations by the combined kernel in circRNA and drug spaces. In order to evaluate the effectiveness of DHANMKF, it was compared with six state-of-the-art methods on a benchmark data set under 5-fold cross-validations (5-CV). Compared with the other methods, DHANMKF obtained the highest AUC. Furthermore, an ablation study was performed to compare the experimental results from different perspectives. Finally, case studies were conducted to demonstrate that the DHANMKF model can be a useful tool for helping with the study of circRNA-drug sensitivity associations in real situations. To the best of our knowledge, DHANMKF is the first algorithm to use dual hierarchical attention networks for the prediction of circRNA-drug sensitivity associations. Our main contributions, differing from previous approaches, are summarized as follows: (1) We classify nodes into two types, i.e., head nodes and tail nodes, based on the degree of the nodes, and then define the types of edges based on the associations between different kinds of nodes. (2) Based on the differences in types of edges, we use dual hierarchical attention networks to extract the information on circRNAs and drugs, use the multi-kernel fusion method to fuse this information, and then use the dual graph regularized least squares method to predict potential circRNA-drug associations. (3) We tested DHANMKF on two datasets, and the results show that multi-relational dual hierarchical attention networks perform better than the other methods in predicting potential circRNA-drug associations. These results can provide new insights for further research on circRNA-drug associations.

## Materials and methods

### Datasets

Two datasets, data271 and data251, were used in this study. Data271 is from Deng et al. [15] and data251 is from Deng et al. [15] and Peng et al. [21]. CircRNA-drug sensitivity associations were collected from the circRic database [14] by Deng et al. [15], where drug sensitivity data were obtained from the GDSC database [22]. After Wilcoxon tests with a false discovery rate $<0.05$, these significant circRNA-drug sensitivity associations were extracted as the data271 dataset, which contains ${N}_{c}=271$ circRNAs, ${N}_{d}=218$ drugs and 4134 circRNA-drug sensitivity associations. Integrating with the dataset of Peng et al. [21], we removed circRNAs with host-gene interaction scores $\le 0.5$ and nodes with a degree of 0. This resulted in the data251 dataset, containing ${N}_{c}=251$ circRNAs, ${N}_{d}=217$ drugs, and 3635 circRNA-drug sensitivity associations. Additional information on these two datasets can be found in the Supplementary file. In our experiment, circRNAs and drugs were represented as two different types of nodes in the network. The node set of $\textbf{N}_{c}$ circRNAs was defined as $\textbf{C}=\{{c}_1, \ldots , {c}_{N_c}\}$. Similarly, the node set of $\textbf{N}_{d}$ drugs was described as $\textbf{D}= \{{d}_1, \ldots , {d}_{N_d}\}$. An adjacency matrix $\textbf{Y} \in R^{{N}_{c} \times {N}_{d}}$ was created for the storage of circRNA-drug associations. In this matrix, ${N}_{c}$ rows represent the number of circRNAs and ${N}_{d}$ columns represent the number of drugs. If circRNA ${c}_{i}$(${1 \le i \le {N}_{c}}$) is associated with drug ${d}_{j}$(${1 \le j \le {N}_{d}}$), $\textbf{Y}_{ij}=1$, otherwise $\textbf{Y}_{ij}=0$. During the training phase, all the $\textbf{Y}_{ij}=1$ are treated as positive samples and the others are treated as negative samples. We randomly masked some positive samples from $\textbf{Y}$ to get $\textbf{Y}_{\textbf{train}}$. In order to calculate the similarity of circRNAs and drugs, the host gene sequences of circRNAs were downloaded from the National Center for Biotechnology Information (NCBI) gene database [23] and the drug structure data were downloaded from NCBI’s PubChem database [24].

### Sequence similarity of host genes of circRNAs

Applying methods similar to those of Deng et al. [15] and Yang et al. [17], we treated the sequence similarity between host genes of circRNAs as the similarity between circRNAs. In this way, the similarity calculation between circRNAs became the sequence similarity calculation between host genes of circRNAs. The sequence similarity between host genes of circRNAs was calculated based on the sequence Levenshtein distance, which was obtained using the ratio function of Python’s Levenshtein package. A similarity matrix $\textbf{CSS} \in R^{{N}_{c} \times {N}_{c}}$ was created for storing the circRNA sequence similarity.

### Structural similarity of drugs

The structure of drugs has a great impact on their function. Therefore, it has become a common practice to measure the similarity of drugs based on their structure. As in previous studies [25, 26], RDKit [27] toolkit and the Tanimoto method were used to calculate the structural similarities between drugs. The specific process was as follows: first, the structural data on several drugs were obtained from the PubChem database. Then, RDKit was used to calculate the topological fingerprint of each drug. After that, the structural similarity between drugs was calculated using the Tanimoto method. Finally, the drugs structural similarity matrix $\textbf{DSS} \in R^{{N}_{d} \times {N}_{d}}$ was derived.

### Gaussian interaction profile kernel similarity for circRNAs and drugs

The Gaussian Interaction Profile (GIP) kernel similarity [28] algorithm is a collaborative filtering algorithm that has been widely used in previous studies for similarity calculation [29, 30], and it helps to obtain topological information on circRNAs and drugs in relational graphs. Therefore, we calculated the GIP kernel similarity for circRNAs and drugs using the circRNA-drug association network. Firstly, based on the assumption that similar circRNAs are more likely to be associated with similar drugs, we utilized a binary vector $\textbf{BI}(c_{i})$, which is the *ith* row of the $\textbf{Y}_{\textbf{train}}$ matrix, representing the associations between circRNAs $c_{i}$ and all drugs in the training matrix of $\textbf{Y}$. Then, the GIP kernel similarity for circRNAs $\textbf{CGS}(c_{i}, c_{j})$ between circRNA $c_{i}$ and $c_{j}$ was calculated as below:

1 $$\begin{aligned} \textbf{CGS}(c_{i}, c_{j}) = exp\left( -\gamma _{c} \Vert \textbf{BI}(c_{i})-\textbf{BI}(c_{j}) \Vert ^2 \right) , \end{aligned}$$

2 $$\begin{aligned} \gamma _{c} = \alpha _{c}/\left( \frac{1}{N_{c}}\sum _{i=1}^{N_{c}} \Vert \textbf{BI}(c_{i})) \Vert ^2 \right) . \end{aligned}$$

Here, $\alpha _{c}$ has been set to 1 referring to [28]’s studies. And similarly, we calculated the GIP of drug $\textbf{DGS}(d_{i}, d_{j})$ between drugs $d_{i}$ and $d_{j}$ as follows:

3 $$\begin{aligned} \textbf{DGS}(d_{i}, d_{j}) = exp\left( -\gamma _{d} \Vert \textbf{BI}(d_{i})-\textbf{BI}(d_{j}) \Vert ^2 \right) , \end{aligned}$$

4 $$\begin{aligned} \gamma _{d} = \alpha _{d}/\left( \frac{1}{N_{d}}\sum _{i=1}^{N_{d}} \Vert \textbf{BI}(d_{i})) \Vert ^2 \right) . \end{aligned}$$

Here, the binary vector $\textbf{BI}(d_{i})$ is the *ith* column of the $\textbf{Y}_{\textbf{train}}$ matrix, representing the associations between drugs $d_{i}$ and all circRNAs in the training matrix of $\textbf{Y}$. $\alpha _{d}$ has been set to 1 referring to [28] studies.

### Integrated similarity for circRNAs and drugs

Inspired by the study of Wang et al. [31], we used a non-linear fusion method to integrate the circRNA similarity and the drug similarity. With circRNA similarity, for example, we first normalized the sequence similarity of host genes of circRNAs using the following formula:

5 $$\begin{aligned} \textbf{CSS}^{\mathbf {'}}(i,j) = \frac{\textbf{CSS}(i,j)}{\sum \limits _{j} \textbf{CSS}(i,j)}. \end{aligned}$$

Then, the *K* Nearest Neighbors (KNN) algorithm was used to measure $\textbf{CSS}$’s local affinity as follows:

6 $$\begin{aligned} \textbf{CKNN1} = \left\{ \begin{array}{ll} \frac{\textbf{CSS}(i,j)}{\sum \limits _{k \in N_{i}^{'}} \textbf{CSS}(i,k)},&{} j\in N_{i}^{'}\\ 0, &{} \text {otherwise}\\ \end{array}\right. . \end{aligned}$$

$N_{i}^{'}$ in Eq. (6) is the set of KNN of $c_{i}$, including $c_{i}$ in $\textbf{CSS}$. This operation is based on the assumption that the higher the local similarity, the more reliable it is. Therefore, the near-end similarity is high while the far-end similarity is set to 0. Similarly, we repeated the process for $\textbf{CGS}$ and then we obtained $\textbf{CGS}^{\mathbf {'}}$ and $\textbf{CKNN2}$. After that, we updated the similarity matrix for each kind of data as follows:

7 $$\begin{aligned} \textbf{CSS}^{\mathbf {'(t+1)}} = \textbf{CKNN1} \times \textbf{CGS}^{\mathbf {'(t)}} \times (\textbf{CKNN1})^{T}. \end{aligned}$$

8 $$\begin{aligned} \textbf{CGS}^{\mathbf {'(t+1)}} = \textbf{CKNN2} \times \textbf{CSS}^{\mathbf {'(t)}} \times (\textbf{CKNN2})^{T}. \end{aligned}$$

After each iteration, $\textbf{CSS}^{\mathbf {'(t+1)}}$ is normalized by formula Eq. (5). Similarly, $\textbf{CGS}^{\mathbf {'(t+1)}}$ performs the same normalization. The iteration does not stop until the convergence condition is met, and the convergence condition is met when the relative change in $\frac{\Vert \textbf{CSS}^{\mathbf {'(t+1)}} - \textbf{CSS}^{\mathbf {'(t)}}\Vert }{\Vert \textbf{CSS}^{\mathbf {'(t)}} \Vert }$ and $\frac{\Vert \textbf{CGS}^{\mathbf {'(t+1)}} - \textbf{CGS}^{\mathbf {'(t)}}\Vert }{\Vert \textbf{CGS}^{\mathbf {'(t)}} \Vert }$ is less than $10^{-6}$. Assuming that the process involves *t* iterations, the overall comprehensive similarity matrix of circRNA can be obtained by Eq. (9) when the iteration ends.

9 $$\begin{aligned} \textbf{S}_{\textbf{c}} = \frac{\textbf{CSS}^{\mathbf {'(t)}}+\textbf{CGS}^{\mathbf {'(t)}}}{2}. \end{aligned}$$

Based on these rules, the similarity matrix $\textbf{S}_{\textbf{c}}$ is an asymmetry matrix. Therefore, we calculated the $\textbf{S}_{\textbf{c}} = \frac{\textbf{S}_{\textbf{c}} + \textbf{S}_{\textbf{c}}^{\textbf{T}}}{2}$ as the circRNA comprehensive similarity matrix. For drugs, we applied the same rules to $\textbf{DSS}$ and $\textbf{DGS}$, then we obtained the comprehensive drug similarity matrix $\textbf{S}_{\textbf{d}}$.

### DHANMKF

Dual Hierarchical Attention Networks (DHAN) were proposed by Zhao et al. [32] in 2022. Comprehensive node representations are learned with intra-type and inter-type attention-based encoders using a hierarchical process based on the bi-typed multi-relational heterogeneous graphs in DHAN. Specifically, DHAN uses two encoders, one to aggregate information on nodes of the same type and the other to aggregate node representations of different type neighbors. Then, the complex structure of the bi-typed multi-relational heterogeneous graph is captured by the model by a hierarchical process and dual-level attention operation. It is worth noting that the association matrix Y of circRNA-drug is a bi-typed single-relation heterogeneous graph. Therefore, in order to fully utilize the extraction ability of DHAN for node embedding, it is necessary to classify the relationships between nodes.

It is well-known that the adjacency matrix describing different objects in the biomedical field is sparse. This means that there are many nodes with small degrees. Histograms of the degree distributions of circRNAs and drugs can be found in the Supplementary file. It can be seen that most of the nodes have small degrees regardless of whether they are circRNA nodes or drug nodes. Intuitively, the biomedical significance of a drug being associated with only a few circRNAs or a drug being associated with many circRNAs are different. Inspired by Liu et al. [33], we categorized the nodes into head nodes and tail nodes according to the value of their degrees. That is, for every node $v \in V$, where *V* is the set of nodes in a graph. $N_{v}$ is denoted by the set of neighboring nodes of *v*, and the number of elements in set $N_{v}$ is defined as the degree of *v*. Here we let $V_h$ and $V_t$ denote the set of head and tail nodes, respectively. For some threshold *K*, we define tail nodes as nodes with a degree not exceeding *K*, i.e., $V_t = \{ v :|N_v |\le K \}$, whereas head nodes are the complement of tail nodes, that is, $V_h = \{ v :|N_v |> K \}$. *K* is treated as a hyperparameter in our study. In this way, the association of circRNAs with drugs in dataset data271 changes from being one type to being the following four types.

1. Association between the head node of circRNA and the head node of the drug.
2. Association between the head node of circRNA and the tail node of the drug.
3. Association between the tail node of circRNA and the head node of the drug.
4. Association between the tail node of circRNA and the tail node of the drug.

Because the node types in the circRNA similarity network and the drug similarity network are the same, that is, they are either all circRNA or all drugs, the following three types of associations will be in these two similarity networks.

1. Associations between the head nodes.
2. Associations between head node and tail node.
3. Associations between tail nodes.

In summary, we represent intra-type relationships and inter-type relationships as $R_{intra}=\{1,2,3\}$ and $R_{inter}=\{1,2,3,4\}$, respectively. Whereas in the data251 dataset, circRNAs were split into two types depending on whether the host gene of the circRNA was associated with a disease or not, which is analogous to splitting circRNAs into head nodes and tail nodes. Thus the same number of edge types can also be obtained, and the definition is more biologically meaningful in this way.

#### Intra-type attention-based encoder

After the computational process above, the associated network of circRNA and drug becomes a bi-type multi-relationship heterogeneous network, given a node pair $(n_i,n_j) \in C$ that are connected via node intra-type relationship $\Phi _k \in R_{intra}^{(c)}=\{1,2,3\}$. Firstly, we initialized the representation matrix of circRNA to $\textbf{H}_0^{c}=\textbf{S}_{c}\textbf{Y}_{train}\textbf{W}_{intra}^c=[\textbf{h}_{1}^T, \dots , \textbf{h}_{N_c}^T]^T$. Where $\textbf{W}_{intra}^c \in R^{N_d \times d^{'}}$ is a learnable parameter $\textbf{H}_0^{c} \in R^{N_c \times d^{'}}$, and $\textbf{h}_{i} \in R^{d^{'}}$(${1 \le i \le {N}_{c}}$) is the feature vector of the node $n_i$. Secondly, self-attention was performed on the circRNA nodes to formulate the importance $e_{ij}^{\Phi _{k}}$ of a specific-relation based node pair $(n_i, n_j )$ as follows:

10 $$\begin{aligned} e_{ij}^{\Phi _{k}} = att_{local}(\textbf{h}_{i}, \textbf{h}_{j};\Phi _k) = LeakyRelu(\textbf{a}_{\Phi _{k}^T} \centerdot [\textbf{h}_{i} || \textbf{h}_{j}]). \end{aligned}$$

Where || denotes the concatenate operation, and $\textbf{a}_{\Phi _{k}^T} \in R^{2d^{'} \times 1}$ denotes the shared node-level attention weight vector under relation $\Phi _{k}$. LeakyRelu is the nonlinearity activation function, which is widely used in attention-based neural networks. In the third step, $e_{ij}^{\Phi _{k}}$ is standardized using the Eq. (11) to facilitate comparison of importance between different nodes.

11 $$\begin{aligned} \alpha _{ij}^{\Phi _{k}} = softmax_{j}(e_{ij}^{\Phi _{k}})=\frac{exp(e_{ij}^{\Phi _{k}})}{\sum _{n_{p} \in N_{intra}^{\Phi _k}(n_i)}exp(e_{ij}^{\Phi _{k}})} \end{aligned}$$

Where $N_{intra}^{\Phi _k}(n_i)$ denotes specific relation-based neighbors of $n_i$, the embedding $\textbf{h}_{1}^{\Phi _k}$ of node $n_i$ under given relation $\Phi _k$ is obtained as follows:

12 $$\begin{aligned} \textbf{h}_{i}^{\Phi _{k}} = LeakyRelu \left( Norm_{\Phi _{k}} \left( \sum _{n_{j} \in N_{intra}^{\Phi _k}(n_i)} \alpha _{ij}^{\Phi _{k}} \centerdot \textbf{h}_{j} \right) \right) . \end{aligned}$$

Where $Norm_{\Phi _{k}}$ denotes the relation-specific layer normalization operation; $\textbf{h}_{i}^{\Phi _{k}}$ is semantic-specific. Therefore, by using Eq. (12) to fuse the aggregated information of nodes with different specific relations, more comprehensive node embeddings can be obtained as follows:

13 $$\begin{aligned} g_{i}^{\Phi _{k}} = \textbf{q}^T \left( \textbf{h}_{i} || \textbf{h}_{i}^{\Phi _{k}} \right) . \end{aligned}$$

Where $\textbf{q} \in R^{2d^{'} \times 1}$ is a trainable parameter. Similar to Eq. (11), we standardize $g_{i}^{\Phi _{k}}$ by using the softmax function as follows:

14 $$\begin{aligned} \beta _{ij}^{\Phi _{k}} = softmax_{k}(g_{i}^{\Phi _{k}})=\frac{exp(g_{i}^{\Phi _{k}})}{\sum _{\Phi _{l} \in N_{intra}^{(c)}}exp(g_{i}^{\Phi _{l}})}. \end{aligned}$$

Here $\beta _{ij}^{\Phi _{k}}$ is used to measure the local importance of intra-relation $\Phi _{k}$. Finally, the intra-type attention-based representation of circRNA node $n_i$ can be obtained as follows:

15 $$\begin{aligned} \textbf{z}_{i}^{c} = \sum _{\Phi _{l} \in N_{intra}^{(c)}} \left( t\beta _{G}^{\phi _l} + (1-t)\beta _{i}^{\phi _l}\right) \centerdot \textbf{h}_{i}^{\Phi _l}. \end{aligned}$$

Where $\textbf{z}_{i}^{c} \in R^{d^{'} \times 1}$, $\beta _{G}^{\phi _l}$ denotes how important intra-type $\Phi _l$ is for all circRNA nodes and can be regarded as a global importance parameter. The global and local importance of the intra-type relationship ${\Phi _l}$ is smoothed by the parameter *t*. Both $\beta _{G}^{\phi _l}$ and *t* can be learned from training. The aggregated information for node $n_i$ under intra-type relation $\Phi _l$ is represented by $\textbf{h}_{i}^{\Phi _l}$. Initialize the representation matrix of drug to $\textbf{H}_0^{d}=\textbf{S}_{d}\textbf{Y}_{train}^T\textbf{W}_{intra}^d$, where $\textbf{W}_{intra}^d \in R^{N_c \times d^{'}}$ is a learnable parameter and $\textbf{H}_0^{d} \in R^{N_d \times d^{'}}$. Using the same process above, we can get the intra-type attention-based representation of drug node $n_i$, which can be represented as $\textbf{z}_{i}^{d} \in R^{d^{'}} \times 1$. Let $\textbf{Z}_1^{c}=[(\textbf{z}_{1}^{c})^T,\dots ,(\textbf{z}_{N_c}^{c})^T]^T$ and $\textbf{Z}_1^{d}=[(\textbf{z}_{1}^{d})^T,\dots ,(\textbf{z}_{N_d}^{d})^T]^T$ respectively represent the first layer output of the intra-type attention-based encoder, that is, the node embedding matrix of circRNAs and drugs. Assuming that the intra-type attention-based encoder has *t* layers, the output of the previous layer is taken as the input of the next layer. Repeating this process can obtain *t* node embedding matrices about circRNA and drugs as follows: $\textbf{Z}_1^{c},\dots ,\textbf{Z}_{t}^{c}$, $\textbf{Z}_1^{d},\dots ,\textbf{Z}_{t}^{d}$.

#### Inter-type attention-based encoder

The purpose of the intra-type attention-based encoder is to learn node embeddings by aggregating the node information of the same type neighbors, while the purpose of the inter-type attention-based encoder is to handle interactions between different types of nodes. Let $n_i \in C$ and $n_j \in D$, respectively. $\textbf{z}_{i}^{c}$ and $\textbf{z}_{j}^{d}$ are the learned representations of the circRNA node $n_i$ and drug node $n_j$ by intra-type attention networks, respectively. The node-level importance $c_{ij}^{\Phi _m}$ can be calculated by Eq. (16) and normalized by Eq. (17) as follows:

16 $$\begin{aligned} c_{ij}^{\Phi _m}&= att_{node} \left( \textbf{z}_{i}^{c}, \textbf{z}_{j}^{c}; \Phi _m \right) \\&=LeakyRelu\left( \textbf{a}_{\Phi _m}^{T}, \centerdot \left[ \textbf{W}_{inter}^{c}\textbf{z}_{i}^{c}||\textbf{W}_{inter}^{d}\textbf{z}_{j}^{d} \right] \right) , \end{aligned}$$

17 $$\begin{aligned} \gamma _{ij}^{\Phi _{m}} = softmax_{j}(c_{ij}^{\Phi _m})=\frac{exp(c_ij^{\Phi _m})}{\sum _{n_{k} \in N_{inter}^{\Phi _m}(n_i)}exp(c_ij^{\Phi _m})}. \end{aligned}$$

$N_{inter}^{\Phi _m}(n_i)$ denotes the neighbors of node $n_i$ under specific inter-relation $\Phi _m$. $\textbf{W}_{inter}^{c}$ and $\textbf{W}_{inter}^{d} \in R^{d^{'} \times d^{'}}$ are two type-specific matrices that map their features $\textbf{z}_{i}^{c}$ and $\textbf{z}_{i}^{d}$ into a common space. $\textbf{a}_{\Phi _m} \in R^{2d^{'}}$ is a learnable weight vector. The relationship embedding of circRNA node $n_i$ can be aggregated from the embeddings of its neighbors of different types(that is, the nodes of a drug), with corresponding coefficients as follows:

18 $$\begin{aligned} \textbf{z}_{i}^{\Phi _{m}} = LeakyRelu \left( Norm_{\Phi _{m}} \left( \sum _{n_{j} \in N_{inter}^{\Phi _m}(n_i)} \gamma _{ij}^{\Phi _{m}} \textbf{W}_{inter}^d \textbf{z}_{j}^{d}\right) \right) . \end{aligned}$$

$Norm_{\Phi _{m}}$ denotes the layer normalization operation related to the inter-type relation. Then, the importance of relation embedding $\textbf{z}_{i}^{\Phi _{m}}$ related to node $n_i$ are obtained by fusing all relational representations by Eq. (19), and it is normalized by Eq. (20) for making relation importance comparable within inter-type relations.

19 $$\begin{aligned} f_{i}^{\Phi _{m}} = \tilde{\textbf{q}}^T \left( \textbf{z}_{i}^{c} || \textbf{z}_{i}^{\Phi _m} \right) , \end{aligned}$$

20 $$\begin{aligned} \epsilon _{i}^{\Phi _{m}} = softmax_{m}\left( f_{i}^{\Phi _{m}}\right) =\frac{exp(f_{i}^{\Phi _{m}})}{\sum _{\Phi _{n} \in N_{inter}}exp(f_{i}^{\Phi _{n}})}. \end{aligned}$$

Finally, the representation $\textbf{u}_i$ of circRNA node $n_i$ is obtained by fusing these relation-specific representations as follows:

21 $$\begin{aligned} \textbf{u}_i^{c} = \sum _{\Phi _{m} \in N_{inter}} \epsilon _{i}^{\Phi _{m}} \centerdot \textbf{z}_{i}^{\Phi _{m}}. \end{aligned}$$

Similarly, we can get the inter-type attention-based representation of drug node $n_j$, which can be represented as $\textbf{u}_{j}^{d}$. Let $\textbf{U}_1^{c}=[(\textbf{u}_{1}^{c})^T,\dots ,(\textbf{u}_{N_c}^{c})^T]^T$ and $\textbf{U}_1^{d}=[(\textbf{u}_{1}^{d})^T,\dots ,(\textbf{u}_{N_d}^{d})^T]^T$ respectively represent the first layer output of the inter-type attention-based encoder, that is, the node embedding matrix of circRNAs and drugs. Assuming the inter-type attention-based encoder has *M* layers, the output of the previous layer is taken as the input of the next layer. Repeating this process can obtain *M* node embedding matrices about circRNA and drugs as follows: $\textbf{U}_1^{c},\dots ,\textbf{U}_{M}^{c}$, $\textbf{U}_1^{d},\dots ,\textbf{U}_{M}^{d}$.

#### Multi-kernel fusion

We can extract multiple embeddings from the intra-type attention-based encoder and the inter-type attention-based encoder that represent the information on circRNA nodes and drug nodes of different types and different relationships. For all the embeddings of circRNA and drug, we used the GIP kernel similarity function to calculate the circRNA and drug kernel matrices in each layer as follows:

22 $$\begin{aligned} \textbf{IC}_{l}(i,j) = exp\left( -\gamma _{l}\Vert \textbf{H}_{c}^{(l)}(i)-\textbf{H}_{c}^{(l)}(j)\Vert ^2 \right) , \end{aligned}$$

23 $$\begin{aligned} \textbf{ID}_{l}(i,j) = exp\left( -\gamma _{l}\Vert \textbf{H}_{d}^{(l)}(i)-\textbf{H}_{d}^{(l)}(j)\Vert ^2 \right) . \end{aligned}$$

Where $\textbf{IC}_{l}\in R^{N_{c} \times N_{c}}$, $\textbf{ID}_{l}\in R^{N_{d} \times N_{d}}$, $\textbf{H}_{c}^{(l)}(i)$ and $\textbf{H}_{d}^{(l)}(i)$ represent the *i*-th row of matrix $\textbf{H}_{c}^{(l)}$ and $\textbf{H}_{d}^{(l)}$ respectively; $\textbf{H}_{c}^{(l)}$ and $\textbf{H}_{d}^{(l)}$ are the *l*-th element of the circRNA embedding matrix set $\textbf{H}_c = \{ \textbf{H}_{0}^{c}, \textbf{Z}_1^{c},\dots ,\textbf{Z}_{t}^{c}, \textbf{U}_1^{c},\dots ,\textbf{U}_{M}^{c} \}$ and the drug embedding matrix set $\textbf{H}_d = \{ \textbf{H}_{0}^{d}, \textbf{Z}_1^{d},\dots ,\textbf{Z}_{t}^{d}, \textbf{U}_1^{d},\dots ,\textbf{U}_{M}^{d} \}$, respectively. $\gamma _{l}$ denotes the corresponding bandwidth, we set $\gamma _{l}=\gamma ,l=1,\cdots ,K+1$, and $\gamma$ is a hyperparameter, and $K+1$ is the number of elements in the circRNA embedding matrix set $\textbf{H}_c$ and the drug embedding matrix set $\textbf{H}_d$.

We integrated all the kernels above with multiple kernel fusion in order to fully utilize the information and improve the performance of predicting circRNA-drug associations, then the final kernel matrices of circRNA and drug were obtained as follows:

24 $$\begin{aligned} \textbf{IC} = \sum _{i=0}^{K}\omega _{i}^{c}\textbf{IC}_{i}, \end{aligned}$$

25 $$\begin{aligned} \textbf{ID} = \sum _{i=0}^{K}\omega _{i}^{d}\textbf{ID}_{i}. \end{aligned}$$

Where $\textbf{IC}\in R^{N_{c} \times N_{c}}$, $\textbf{ID}\in R^{N_{d} \times N_{d}}$, $\omega _{i}^{m}=\frac{1}{K+1}$, and $\omega _{i}^{d}=\frac{1}{K+1}$ are the corresponding weight of circRNA kernels and drug kernels, respectively.

### Dual Laplacian regularized least squares model

Inspired by previous studies [34] and [35], the Dual Laplacian Regularized Least Squares (DLapRLS) method was adopted by us to predict circRNA-drug associations. Overfitting was avoided by adding graph regularization with DLapRLS. Thus, the loss function can be defined as follows:

26 $$\begin{aligned} \text {min} J&= \Vert \textbf{IC}\varvec{\alpha }_{c}+ \left( \textbf{ID}\varvec{\alpha }_{d}\right) ^T-2\textbf{Y}_{train}\Vert _{F}^{2}\\&\quad +\phi _{c}tr\left( \varvec{\alpha }_{c}^{T}\textbf{L}_{c}\varvec{\alpha }_{c}\right) +\phi _{d}tr\left( \varvec{\alpha }_{d}^{T}\textbf{L}_{d}\varvec{\alpha }_{d}\right) . \end{aligned}$$

Where $\Vert \cdot \Vert _{F}$ is the Frobenius norm, $\varvec{\alpha }_c$ and $\varvec{\alpha }_{d}^{T}\in R^{N_{c}\times N_{d}}$ are learnable matrices, $\phi _{c}$ and $\phi _{d}$ are regularization parameters.; $\textbf{L}_{c} \in R^{N_{c}\times N_{c}}$ and $\textbf{L}_{d} \in R^{N_{d} \times N_{d}}$ are the normalized Laplacian matrices, as follows:

27 $$\begin{aligned} \textbf{L}_{c} = \textbf{V}_{c}^{-1/2}\varvec{\Delta }_{c}\textbf{V}_{c}^{-1/2}, \varvec{\Delta }_{c}=\textbf{V}_{c}-\textbf{IC}, \end{aligned}$$

28 $$\begin{aligned} \textbf{L}_{d} = \textbf{V}_{d}^{-1/2}\varvec{\Delta }_{d}\textbf{V}_{d}^{-1/2}, \varvec{\Delta }_{d}=\textbf{V}_{d}-\textbf{ID}. \end{aligned}$$

Where $\textbf{V}_{c}=\sum _{i=1}^{N_{c}}\textbf{IC}$ and $\textbf{V}_{d}=\sum _{i=1}^{N_{d}}\textbf{ID}$ are diagonal degree matrix. Finally, the prediction $\hat{\textbf{F}}$ for circRNA-drug associations from $\textbf{IC}$ and $\textbf{ID}$ is obtained as follows:

29 $$\begin{aligned} \hat{\textbf{F}} = \frac{\textbf{IC}\varvec{\alpha }_{c}+\left( \textbf{ID}\varvec{\alpha }_{d}\right) ^{T}}{2}. \end{aligned}$$

### Training

Except for parameters $\varvec{\alpha }_{c}$ and $\varvec{\alpha }_{d}$, the parameters of our model are updated by Adam [36]. The parameters of $\varvec{\alpha }_{c}$ and $\varvec{\alpha }_{d}$ are updated by calculating the partial derivatives for the parameters of DLapRLS. The specific calculation process is as follows: we first assume that $\varvec{\alpha }_{d}$ is a constant matrix when $\varvec{\alpha }_{d}$ is optimized. Thus, the partial derivative of the loss function Eq. (26) with respect to $\varvec{\alpha }_{c}$ can be calculated as follows:

30 $$\begin{aligned} \frac{\partial J}{\partial \varvec{\alpha }_{c}} = 2\textbf{IC}\left( \textbf{IC}\varvec{\alpha }_{c} + \left( \textbf{ID}\varvec{\alpha }_{d}\right) ^T-2\textbf{Y}_{train}\right) +2\phi _{c}\textbf{L}_{c}\varvec{\alpha }_{c} \end{aligned}$$

Let $\frac{\partial J}{\partial \varvec{\alpha }_{c}} = 0$, then $\varvec{\alpha }_{c}$ can be obtained as follows:

31 $$\begin{aligned} &\left( \textbf{IC}+\phi _{c}\textbf{L}_{c}\right) \varvec{\alpha }_{c}=\textbf{IC}\left[ 2\textbf{Y}_{train}-\varvec{\alpha }_{d}^{T}\textbf{ID}^{T}\right] ,\\&\varvec{\alpha }_{c}=\left( \textbf{IC}+\phi _{c}\textbf{L}_{c}\right) ^{-1}\textbf{IC}\left[ 2\textbf{Y}_{train}-\varvec{\alpha }_{d}^{T}\textbf{ID}^{T}\right] . \end{aligned}$$

Similarly, the partial derivative of the loss function Eq. (26) with respect to $\varvec{\alpha }_{d}$ can be calculated as follows:

32 $$\begin{aligned} \frac{\partial J}{\partial \varvec{\alpha }_{d}} = 2\textbf{ID}\left( \textbf{ID}\varvec{\alpha }_{d} + \left( \textbf{IC}\varvec{\alpha }_{c}\right) ^T-2\textbf{Y}_{train}^{T}\right) +2\phi _{d}\textbf{L}_{d}\varvec{\alpha }_{d}. \end{aligned}$$

Same as above, we let $\frac{\partial J}{\partial \varvec{\alpha }_{d}} = 0$, and then $\varvec{\alpha }_{d}$ can be obtain as follows:

33 $$\begin{aligned}{} & {} \left( \textbf{ID}+\phi _{d}\textbf{L}_{d}\right) \varvec{\alpha }_{d}=\textbf{ID}\left[ 2\textbf{Y}_{train}^{T}-\varvec{\alpha }_{c}^{T}\textbf{IC}^{T}\right] ,\nonumber \\{} & {} \varvec{\alpha }_{d}=\left( \textbf{ID}+\phi _{d}\textbf{L}_{d}\right) ^{-1}\textbf{ID}\left[ 2\textbf{Y}_{train}^{T}-\varvec{\alpha }_{c}^{T}\textbf{IC}^{T}\right] . \end{aligned}$$

**Fig. 1:**
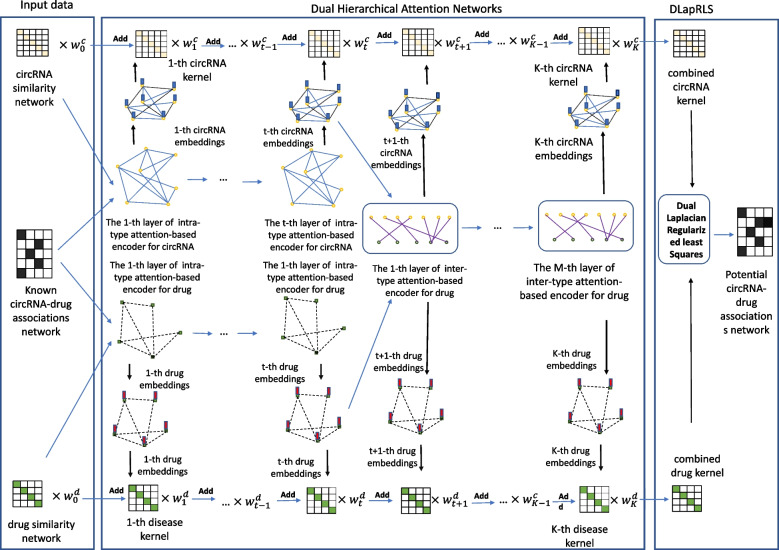
The overview of our proposed method

$\varvec{\alpha }_{c}$ and $\varvec{\alpha }_{d}$ were randomly initialized at the beginning of our model training, and then they were calculated by Eqs. (31) and (33) directly in each iteration, while other parameters were optimized by Adam. The flowchart of our model is shown in Fig. 1. All experimentally verified circRNA-drug associations were treated as positive samples, and the unknown circRNA-drug associations were treated as negative samples, similar to the work of Deng et al. [15] and Yang et al. [17]. Then, the same number of negative samples were randomly selected from all the the unknown circRNA-drug associations. Finally, the same number of positive and negative samples were selected for training.

## Results

### Implementation details and performance evaluation

The model used in this study was implemented based on PyTorch and PyG, and we evaluated the predictive performance of our model using 5-fold cross-validation (5CV). The training epochs were set to 40, the learning rate to 0.05 and the weight decay to 0.01. The number of layers for both the intra-type attention-based encoder and the inter-type attention-based encoder were set to 1 and the output dimensions were both set to 16. The thresholds for distinguishing the head and tail nodes of circRNAs and drugs were set at 27 and 39, respectively. Multi-headed attention was set to 5, and the remaining hyperparameters were set as follows: $\phi _{c}=\phi _{d}=\frac{1}{120}$, $\gamma _c=\gamma _d=\gamma =\frac{1}{75}$.

During evaluation, we randomly divided all the samples into 5 folds. Four of these folds were used as a training set while the remaining fold was treated as a test set. Seven metrics are used to compare model performance: AUC, AUPR, Accuracy, Precision, Recall, F1-Score, and Specificity. It is well-established that improved model performance is reflected by higher AUC and AUPR values. F1-Score is the average of accuracy and recall, while specificity measures the ability of the classifier to correctly identify negative cases.

### Performance comparison with other methods under 5-CV

The current computational methods for predicting circRNA-drug sensitivity associations are restricted. We found that GATECDA [15] and MNGACDA [17] are specifically designed for predicting circRNA-drug sensitivity associations. Thus, like Ref. [15] and Ref. [17], we compared our model with seven state-of-the-art models from different domains, namely MNGACDA [17], GATECDA [15], MINIMDA [37], LAGCN [38], MMGCN [39], and GANLDA [40] . Brief descriptions of these models are provided below:

- MNGACDA [17] : a computational framework for predicting circRNA-drug sensitivity associations. This model uses multimodal networks to learn the embedded representations of circRNAs and drugs, then captures the internal information between nodes in the networks with node-level attention Graph Auto-Encoder.
- GATECDA [15] : a computational model based on Graph Attention Auto-encoder (GATE) for predicting circRNA-drug sensitivity associations.
- MKGCN [35] : a computational model based on GCN and MKL for predicting microbe-drug associations.
- MINIMDA [37] : a method of predicting miRNA-disease associations by constructing integrated similarity networks and using multimodal networks to obtain embedding representations of miRNAs and diseases. These representations are then fed into a multilayer perceptron for prediction.
- LAGCN [38] : LAGCN integrates various associations into a heterogeneous network, learns embeddings of drugs and diseases by Graph Convolution operations, and then combines multiple layers by using an attention function.
- MMGCN [39] : MMGCN differs from simple multisource integration in that it uses a GCN encoder to obtain miRNA and disease features in different similarity views and enhances the learned representations for association prediction by using multichannel attention that adaptively learns the importance of different features.
- GANLDA [40] : this method combines heterogeneous data of lncRNA and disease as original features and reduces noise by using Principal Component Analysis (PCA). Then the Graph Attention Network is used to extract information from the features. Finally, a multi-layer perceptron is used to predict lncRNA-disease associations.

The prediction performance of each method was evaluated by a 5CV experiment using the same settings and optimal parameters recommended in their respective studies. From Table 1, it can be seen that DHANMKF achieved the highest AUC and AUPR values. This indicates that DHANMKF performed better overall compared to the other models.

**Table 1:** Performance comparison based on five-fold cross-validation

Method	F1-Score	Accuracy	Recall	Specificity	Precision	AUC	AUPR
Dataset: data271							
DHANMKF(our)	**0.8500**	**0.8520**	0.8648	**0.8636**	**0.8618**	**0.9178**	**0.9262**
MNGACDA [17]	0.8472	0.8424	**0.8723**	0.8155	0.8247	0.9139	0.9209
GATECDA [15]	0.8224	0.8186	0.8404	0.7966	0.8054	0.8873	0.8915
MKGCN [35]	0.8230	0.8208	0.8221	0.8193	0.8350	0.8768	0.8984
MMGCN [39]	0.8190	0.8183	0.8231	0.8135	0.8156	0.8766	0.8664
MINIMDA [37]	0.7988	0.7901	0.8331	0.7472	0.7684	0.8562	0.8534
LAGCN [38]	0.7900	0.7786	0.8338	0.7233	0.7516	0.8505	0.8478
GANLDA [40]	0.7936	0.7822	0.8384	0.7259	0.7542	0.8517	0.8468
Dataset: data251							
DHANMKF(our)	**0.8597**	**0.8588**	**0.8655**	**0.8521**	**0.8552**	**0.9263**	**0.9286**
MNGACDA [17]	0.8444	0.8429	0.8528	0.8330	0.8362	0.9136	0.9211
MKGCN [35]	0.8253	0.8247	0.8285	0.8208	0.8222	0.8941	0.9090
MMGCN [39]	0.8065	0.8120	0.7836	0.8404	0.8308	0.8842	0.9049
GATECDA [15]	0.8336	0.8287	0.8225	0.8354	0.8451	0.8802	0.9034
MINIMDA [37]	0.8053	0.7950	0.8476	0.7424	0.7670	0.8575	0.8502
LAGCN [38]	0.7880	0.7857	0.7966	0.7748	0.7796	0.8540	0.8615
GANLDA [40]	0.7883	0.7956	0.8089	0.7823	0.7880	0.8539	0.8551

### Evaluation of parameters

The prediction performance of DHANMKF is affected by various parameter values. The parameters of DHANMKF can be divided into four parts: the parameters in the inter-type attention-based encoder and the intra-type attention-based encoder, bandwidth parameter $\gamma$ in MKF, regularization parameters ($\phi _c$ and $\phi _d$) in DLapRL, and degree threshold parameters ($K_c$ and $K_d$) for distinguishing circRNA and drug nodes as head and tail nodes. Here, the process of parameter evaluation is demonstrated using data271 as the baseline dataset.The parameter settings of DHANMKF on the data251 dataset have been put into the Supplementary file.

#### Optimizable parameters in the intra-type attention-based encoder and the inter-type attention-based encoder

 

**Fig. 2:**
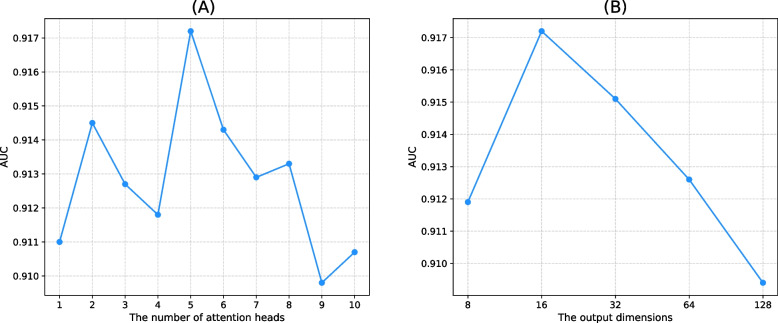
DHANMKF’s attention heads and output dimensions

**Fig. 3:**
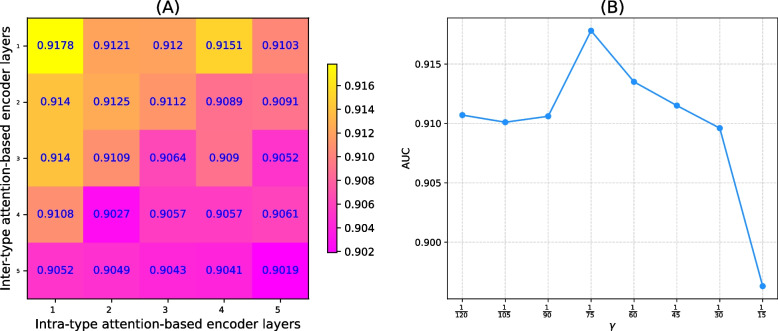
DHANMKF’s layers and $\gamma$

- *Learning rate and its weight decay.* Learning rate and its weight decay are the same in the intra-type attention-based encoder and the inter-type attention-based encoder. Based on the research conducted by Zhao et al. [32], we set them as 0.05 and 0.01 respectively.
- *Dropout and number of model training epochs.* We selected the dropout to be $\{0.02,0.021,\dots ,0.03$}. When the value of the dropout is 0.026, the model performance reaches its optimum, and with an increase in the value of the dropout, the model performance gradually declines. The loss of DHANMKF started converging at 40 training epochs, so the number of epochs for our model was set to 40.
- *The number of attention heads.* To have a more powerful representation learning capacity, the multi-head attention mechanism was incorporated into the model. This parameters was tuned using 5CV. As shown in Fig. 2A, when the number of attention heads is equal to 5, the model performance reaches its optimum.
- *The output dimensions.* We analyzed the output dimensions of the intra-type attention-based encoder and inter-type attention-based encoder, as shown in Fig. 2B. When the output dimension was 16, the AUC performance was best.
- *The number of layers of the intra-type attention-based encoder and the inter-type attention-based encoder.* As shown in Fig. 3A, when the number of layers of the intra-type attention-based encoder and the inter-type attention-based encoder are both 1, the AUC of DHANMKF reaches its optimal value.

#### Optimizable parameters in MKF and DLapRL

 

**Fig. 4:**
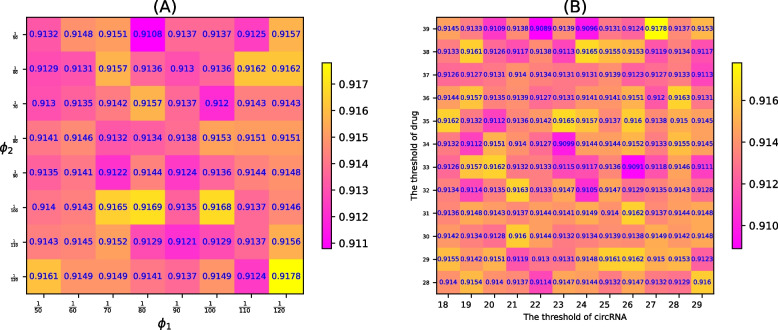
$\phi _1$ and $\phi _2$ in DLapRL, the thresholds for the circRNA node and drug node

- The bandwidth parameter $\gamma$ in MKF is actually the $\frac{1}{2\sigma ^2}$ of the Gaussian kernel function, that is, $\gamma =\frac{1}{2\sigma ^2}$. Parameter $\sigma$ determines the smoothness of the Gaussian filter. The larger $\sigma$ is, the smoother it is. Therefore, by adjusting $\gamma$, a compromise can be reached between over-smoothing and under-smoothing. As shown in Fig. 3B, when $\gamma = \frac{1}{75}$, the AUC of DHANMKF reaches its optimal value.
- The parameters $\phi _c$ and $\phi _d$ play a regulating role in DLapRL, and they can be adjusted to balance underfitting and overfitting. From Fig. 4A, we can see that the AUC of the model reaches its maximum when $\phi _c$ and $\phi _d$ are both $\frac{1}{120}$.

#### Optimization of head and tail node thresholds

The threshold of the head and tail nodes can adjust the number of head and tail nodes and the number of associated types, thus affecting the embedding of corresponding nodes. As shown in Fig. 4B, the maximum AUC value of the model is achieved when the thresholds of the circRNA node and drug node are 27 and 39, respectively.

### Ablation tests

Ablation experiments were conducted from two perspectives: 1. Analyzing the importance of the intra-type attention-based encoder and the inter-type attention-based encoder; 2. Analyzing the effects of multiple relationships. Therefore, we constructed three ablation experiments. The first one is called DHANMKF-intra, which means that DHANMKF removes the embedding produced by the intra-type attention-based encoder when doing Multi-Kernel Fusion. The second one is called DHANMKF-inter, which means that DHANMKF removes the embedding produced by the inter-type attention-based encoder when doing multi-core fusion. The third one is called DHANMKF-multi, which means that the model no longer divides the relationships between nodes into multiple categories.

Table 2 shows the comparison results of the 5CV. From Table 2 we can see that DHANMKF performs better than all other models. This shows that: (1) Compared with DHANMKF-intra and DHANMKF-inter, DHANMKF performs better, which means that the embeddings produced by the intra-type attention-based encoder and the inter-type attention-based encoder improve the performance of the model. (2) Compared with DHANMKF-multi, DHANMKF can generate node embeddings corresponding to different relationships between nodes. In summary, there are two main reasons why DHANMKF can outperform other models. The first reason is that our model can fully capture the complex structures of the bi-typed multi-relational heterogeneous graphs. The second reason is that biological networks in reality are sparse, so it is reasonable to divide the nodes in biological networks into head nodes and tail nodes for analysis.

**Table 2:** Ablation experiment

Model	Dataset: data271		Dataset: data251	
	AUC	AUPR	AUC	AUPR
DHANMKF	**0.9178**	**0.9262**	**0.9263**	**0.9286**
DHANMKF-intra	0.9143	0.9238	0.9230	0.9245
DHANMKF-inter	0.9150	0.9228	0.9238	0.9227
DHANMKF-multi	0.9131	0.9226	0.9186	0.9212

## Case studies

To further evaluate the predictive performance of our model, we selected two drugs, PAC-1 and Vorinostat, for case studies. Similar to Deng et al. [15] and Yang et al. [17], we used the circRNA-drug associations in the GDSC database as the training set and those in the CTRP database as the testing set. For each drug, we chose the top 20 circRNAs with the highest predicted scores from our model’s circRNA-drug association prediction outputs for validation.

PAC-1 is the first known small molecule drug that directly activates procaspase-3 to caspase-3 [41]. It not only enhances procaspase-3 activity but also induces cancer cell apoptosis. Vitro experiments have shown that PAC-1 exhibits cytotoxicity against lymphoma, multiple myeloma, and many other cancer cells [42]. Currently, PAC-1 has been used in clinical trials for the treatment of various tumors, including but not limited to lymphoma, melanoma, solid tumors, breast cancer, and lung cancer [43]. As shown in Table 3, among the top 20 circRNAs predicted by our method to be associated with PAC-1, 16 have been identified in CTRP.

**Table 3:** Top 20 circRNAs related to PAC-1 predicted by DHANMKF

Ranking	circRNA	Evidence	Ranking	circiRNA	Evidence
1	SPARC	CTRP	11	MEF2D	CTRP
2	ARID1B	CTRP	12	PEA15	CTRP
3	LTBP4	Nonsignificant	13	PTMS	CTRP
4	VIM	CTRP	14	CRIM1	CTRP
5	POLR2A	CTRP	15	TCOF1	Nonsignificant
6	SPINT2	Nonsignificant	16	ANP32B	CTRP
7	CTTN	CTRP	17	ENO2	Nonsignificant
8	ASPH	CTRP	18	NCL	CTRP
9	COL1A2	CTRP	19	COL1A1	CTRP
10	THBS1	CTRP	20	ADPGK	CTRP

Belinostat is a small-molecule hydroxamate-type inhibitor that can inhibit the activity of class I, II and IV histone deacetylase enzymes. It has been used to treat relapsed or refractory peripheral T-cell lymphoma [44]. Table 4 shows that 17 of the top 20 circRNAs predicted by our method have been confirmed in circRic.

**Table 4:** Top 20 circRNAs related to Belinostat predicted by DHANMKF

Ranking	circRNA	Evidence	Ranking	circiRNA	Evidence
1	ASPH	CTRP	11	CTSD	CTRP
2	CTTN	CTRP	12	MEF2D	CTRP
3	THBS1	CTRP	13	ILF3	CTRP
4	CRIM1	CTRP	14	FBLN1	CTRP
5	ANXA2	CTRP	15	PHF21A	CTRP
6	LTBP3	CTRP	16	MYC	CTRP
7	PTMS	Nonsignificant	17	GSE1	CTRP
8	POLR2A	Nonsignificant	18	PEA15	CTRP
9	MGAT4B	CTRP	19	COL18A1	CTRP
10	KRT19	Nonsignificant	20	NOP53	CTRP

In order to demonstrate the performance of DHANMKF in predicting the potential association between new drugs and circRNA, we chose two drugs for *ab initio* testing, both of which had only one known circRNA-drug association. During the training phase, we removed the unique association between these two drugs and circRNA. At this point, these two drugs were not associated with any circRNAs and were treated as new drugs during training. These two drugs were Bortezomib and MS-275 (Entinostat). Bortezomib is a novel proteasome inhibitor with potent chemo/radio-sensitizing effects that can overcome the traditional resistance of tumors when used in combination with chemotherapy [45]. In addition, existing clinical applications have shown that Bortezomib can improve clinical outcomes in the treatment of hematologic malignancies [46].

MS-275, also known as Entinostat, is effective in human leukemia cells and lymphoma cells. It can reduce the level of Bcl-XL in cells, induce p21 protein expression, cause cell cycle arrest (G1 phase), and induce cell apoptosis [47]. In addition, when used in combination with other drugs, entinostat can enhance the activity of some anticancer drugs, including Rituximab, Gemcitabine, Doxorubicin, Sorafenib and Bortezomib. Currently, Entinostat is undergoing phase III clinical trials and its clinical data shows that it has great potential for treating breast cancer [48].

As shown in Table 5, 6 of the top 10 predicted circRNAs associated with Bortezomib have been confirmed in circRic, and 7 of the top 10 circRNAs related to MS-275 have been confirmed in circRic.

**Table 5:** The Top 10 predicted circRNAs associated with the two new drugs Bortezomib and MS-275

Bortezomib			MS-275		
Ranking	circRNA	Evidence	Ranking	circiRNA	Evidence
1	POLR2A	Nonsignificant	1	POLR2A	Nonsignificant
2	MEF2D	CTRP	2	CTTN	CTRP
3	VIM	Nonsignificant	3	ASPH	CTRP
4	CRIM1	CTRP	4	ANP32B	CTRP
5	THBS1	CTRP	5	SPINT2	Nonsignificant
6	ADPGK	CTRP	6	CRIM1	CTRP
7	TCOF1	Nonsignificant	7	PTMS	CTRP
8	ENO2	Nonsignificant	8	VIM	Nonsignificant
9	FBLN1	CTRP	9	THBS1	CTRP
10	PEA15	CTRP	10	FBLN1	CTRP

## Conclusions

Recent research over the past twenty years has shown that circRNA plays an important role in drug sensitivity. Therefore, predicting the potential association between circRNA and drug sensitivity can be helpful in drug development and utilization, thus benefiting patients. In this study, we proposed a method, based on intra-type attention and inter-type attention called DHANMKF, for discovering potential circRNA-drug sensitivity associations. To verify the effectiveness of the model, DHANMKF was compared with six state-of-the-art methods based on 5CV on benchmark datasets. The results showed that DHANMKF achieved the best performance. In addition, to further evaluate the ability of the model to discover new drugs, a case study was conducted and the model’s prediction results were validated using an independent database. The validation results clearly demonstrate that DHANMKF is an effective tool for predicting new circRNA-drug sensitivity associations.

The results show that our model outperforms the baseline models. We believe the main reasons are the following: (1) We classify the nodes into head and tail nodes, which in turn defines the types of edges connecting these two types of nodes. This allows our model to extract node embeddings from the circRNA-drug heterogeneous graph based on different types of edges. (2) The intra-type attention-based encoder can efficiently aggregate information from nodes of the same type. (3) The inter-type attention-based encoder adequately extracts node representations from different types of nodes . (4) The MKL method fuses the multi-relational heterogeneous graph information captured by the two encoders in order to improve the overall performance of the model. In future studies, we plan to integrate more biomedical data, in order to generate more comprehensive circRNA and drug kernels and further improve model performance. Currently, there are few studies that use computational methods to predict potential associations between circRNA and drug sensitivity, so further investigation in this field is merited.

### Supplementary Information

**Additional file 1.**

## Data Availability

The data sets and source codes used in this study are freely available at https://github.com/cuntjx/DHANMKF.
