# Supplementary material for: Inferring circRNA-drug sensitivity associations via dual hierarchical attention networks and multiple kernel fusion
Source: BMC Genomics. 2023 Dec 21;24:796. doi: 10.1186/s12864-023-09899-w (PMC10734204; doi:10.1186/s12864-023-09899-w)
Supplement: Supplementary file 1 — Additional file 1. [file 12864_2023_9899_MOESM1_ESM.docx]

**Supplementary document of “Inferring circRNA-drug sensitivity associations via dual hierarchical attention networks and multiple kernel fusion”**

**1 Histograms of the degree distributions of circRNAs and drugs(data271)**

**
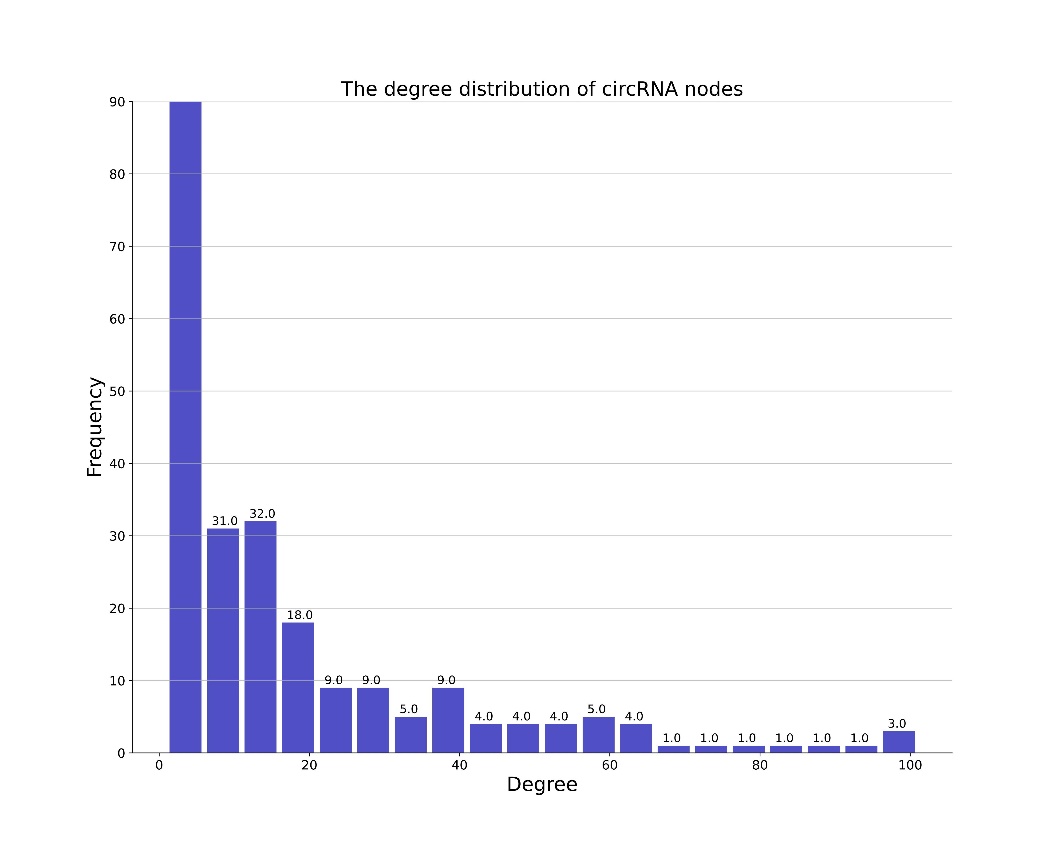
**

S1 Figure: The degree distribution of circRNA nodes

**
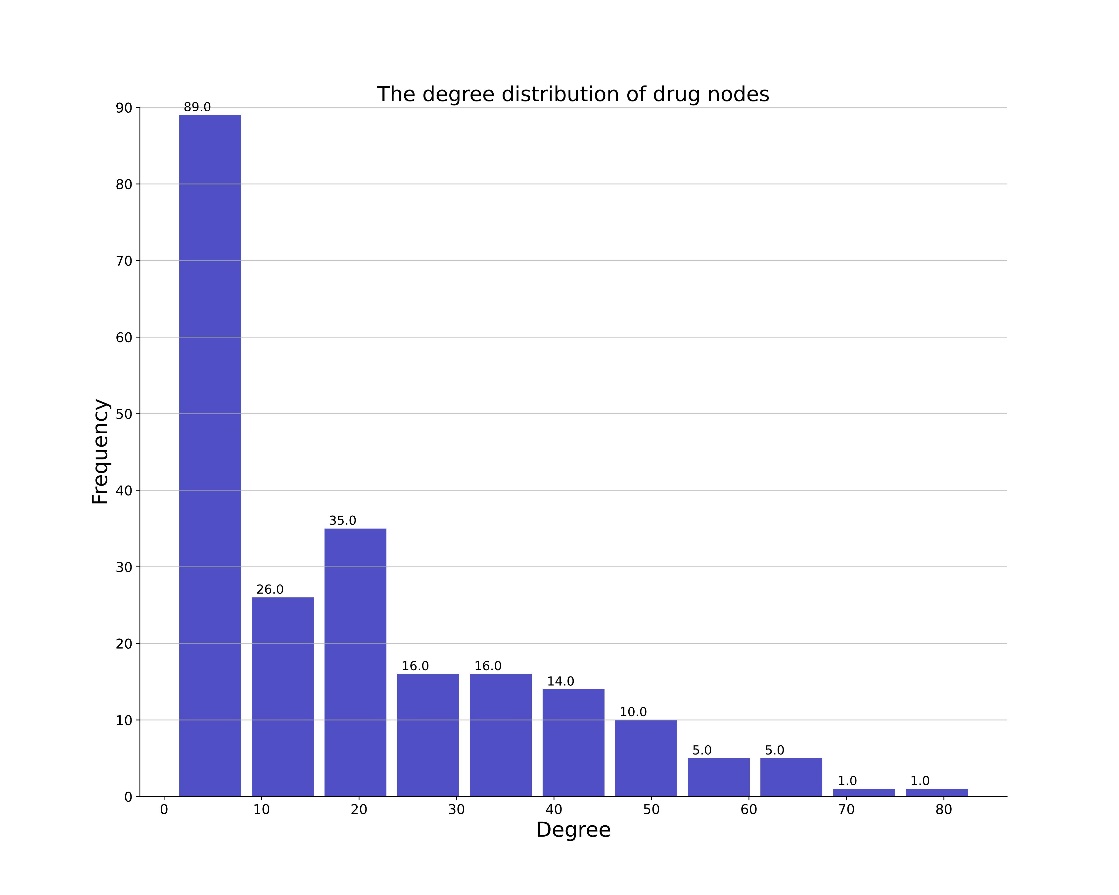
**

S2 Figure: The degree distribution of drug nodes

**2** **Basic characteristics of the two datasets data271 and 251**

S1 Table: The basic characteristics of the data271

|  | Name | Number |
| --- | --- | --- |
| Total | CircRNA | 271 |
|  | Drug | 218 |
|  | Associations | 4134 |
| Min degree | CircRNA | 1 |
|  | Drug | 1 |
| Average degree | CircRNA | 15.25 |
|  | Drug | 18.93 |
| Max degree | CircRNA | 101 |
|  | Drug | 83 |

S2 Table: The basic characteristics of the data251

|  | Name | Number |
| --- | --- | --- |
| Total | CircRNA | 251 |
|  | Drug | 217 |
|  | Associations | 3935 |
| Min degree | CircRNA | 1 |
|  | Drug | 1 |
| Average degree | CircRNA | 15.68 |
|  | Drug | 18.13 |
| Max degree | CircRNA | 101 |
|  | Drug | 78 |

**3 AUC and AUPR of eight different models on data271 dataset**


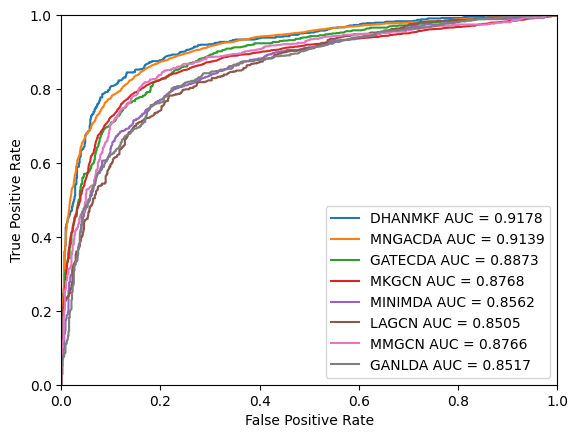


S3 Figure: AUC of different models on data271 dataset


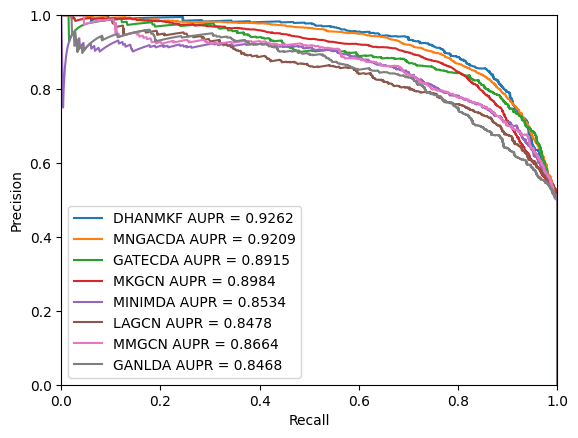


S4 Figure: AUPR of different models on data271 dataset

**4 AUC and AUPR of eight different models on data251 dataset**


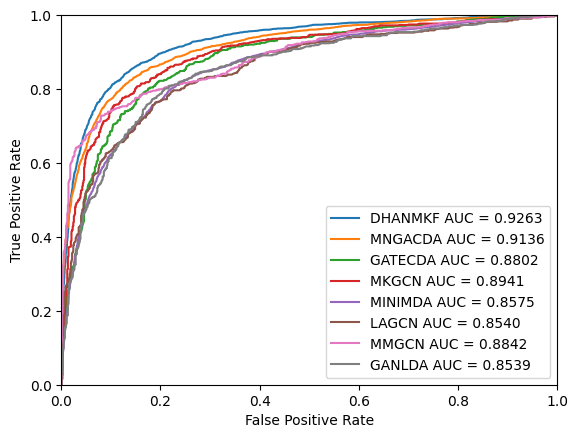


S5 Figure: AUC of different models on data251 dataset


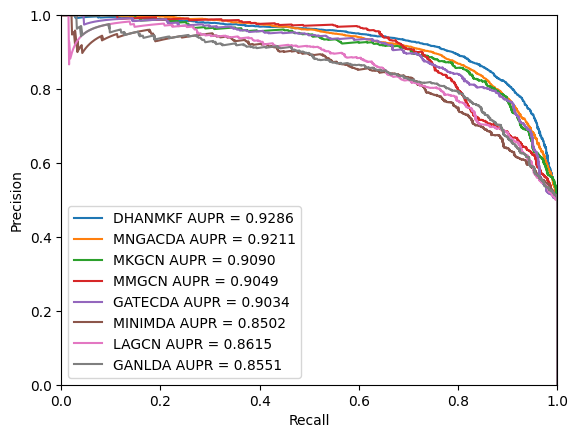


S6 Figure: AUPR of different models on data251 dataset

**5 Parameters Setting of DHANMKF**

S3 Table: Parameters Setting of DHANMKF

| Parameters | data271 | data251 |
| --- | --- | --- |
| epoch | 40 | 40 |
| learning rate | 0.05 | 0.518 |
| weight decay | 0.01 | 0.01 |
| $\phi_{c}$ | $\frac{1}{120}$ | $\frac{1}{100}$ |
| $\phi_{d}$ | $\frac{1}{120}$ | $\frac{1}{100}$ |
| $\gamma_{c}$ | $\frac{1}{75}$ | $\frac{1}{75}$ |
| $\gamma_{d}$ | $\frac{1}{75}$ | $\frac{1}{75}$ |
| $K_{c}$ | 18 | 18 |
| $K_{d}$ | 36 | 37 |
| dropout | 0.026 | 0.026 |
| attention heads | 5 | 5 |
| layers of intra-type attention-based encode | 1 | 1 |
| layers of inter-type attention-based encode | 1 | 1 |
